# Multi-Omics Profiling of the Tumor Microenvironment: Paving the Way to Precision Immuno-Oncology

**DOI:** 10.3389/fonc.2018.00430

**Published:** 2018-10-05

**Authors:** Francesca Finotello, Federica Eduati

**Affiliations:** ^1^Biocenter, Division for Bioinformatics, Medical University of Innsbruck, Innsbruck, Austria; ^2^Department of Biomedical Engineering, Eindhoven University of Technology, Eindhoven, Netherlands

**Keywords:** tumor-infiltrating immune cells, bioinformatics, tumor microenvironment, multi-omics profiling, next-generation sequencing, systems biology, systems immunology, immuno-oncology

## Abstract

The tumor microenvironment (TME) is a multifaceted ecosystem characterized by profound cellular heterogeneity, dynamicity, and complex intercellular cross-talk. The striking responses obtained with immune checkpoint blockers, i.e., antibodies targeting immune-cell regulators to boost antitumor immunity, have demonstrated the enormous potential of anticancer treatments that target TME components other than tumor cells. However, as checkpoint blockade is currently beneficial only to a limited fraction of patients, there is an urgent need to understand the mechanisms orchestrating the immune response in the TME to guide the rational design of more effective anticancer therapies. In this Mini Review, we give an overview of the methodologies that allow studying the heterogeneity of the TME from multi-omics data generated from bulk samples, single cells, or images of tumor-tissue slides. These include approaches for the characterization of the different cell phenotypes and for the reconstruction of their spatial organization and inter-cellular cross-talk. We discuss how this broader vision of the cellular heterogeneity and plasticity of tumors, which is emerging thanks to these methodologies, offers the opportunity to rationally design precision immuno-oncology treatments. These developments are fundamental to overcome the current limitations of targeted agents and checkpoint blockers and to bring long-term clinical benefits to a larger fraction of cancer patients.

## The tumor-immune paradigm shift

In recent years, cancer immunotherapy has revolutionized the treatment of human malignancies: from directly killing tumor cells, to supporting the body's own immune system in the fight against cancer. So far, immune checkpoint blockers (ICBs), i.e., monoclonal antibodies targeting immune-cell regulators to boost antitumor immunity, represent the most successful treatment regimens for solid cancers. ICBs targeting the cytotoxic T-lymphocyte-associated protein 4 (CTLA-4), the programmed cell death protein 1 (PD-1) and its ligand (PD-L1) have shown unprecedented durable responses and are now part of the standard of care for patients with different cancer types (1). However, as ICBs are ineffective for most patients (2, 3), there is a pressing need to elucidate the mechanisms taking place in the tumor microenvironment (TME).

The TME is a complex ecosystem composed of various cell types, their secreted products (e.g., cytokines, chemokines), and other non-cellular components of the extracellular matrix (ECM)

(4, 5). Tumor-infiltrating immune cells play a pivotal role in tumor control and response to therapy (6–8). Cytotoxic CD8^+^ T cells are the primary effectors of natural and therapy-induced anticancer immunity, as they can specifically recognize and kill malignant cells displaying neoantigens (i.e., tumor-specific antigens generated from the expression of mutated genes) (9). But immune cells can also induce immunosuppression and support tumor growth, as in the case of regulatory T (T_reg_) cells, M2 macrophages, and myeloid-derived suppressor cells (MDSCs) (6, 7, 10, 11). Also fibroblasts, the major constituents of the tumor stroma, take part actively to the tumor-immune cell crosstalk. While normal fibroblasts counteract tumor growth, some subsets of cancer associated fibroblasts (CAFs) have been associated with increased cancer cell proliferation and invasion, drug resistance, and reduced anti-tumor immunity (12).

## Dissecting the tumor microenvironment from bulk *Omics* data

The tumor-immune paradigm shift that has revolutionized the oncology field has been also mirrored by bioinformatics. *Omics* data, originally used to perform tumor-centric analyses, are now mined to extract additional features describing the cellular and molecular heterogeneity of the TME and to disentangle tumor-immune cell interactions.

RNA sequencing (RNA-seq) data can be used alone or in combination with whole-exome or whole-genome sequencing data to predict patient-specific cancer neoantigens arisen from somatic mutations, indels, gene fusions, or alternatively spliced transcripts (13–16). Putative neoantigens, which might elicit an anticancer response, can be predicted computationally through three main steps: (1) Prediction of peptides originated from the expression of transformed genes; (2) Reconstruction of patients' Human Leukocyte Antigen (HLA) alleles; (3) Identification of peptides binding to the patients' HLA alleles. Using this approach, two recent studies (17, 18) developed effective personalized, neoantigen-based vaccines for melanoma patients in phase I clinical trial. However, the potential of these strategies is still curtailed by the limited performance of the algorithms for predicting peptide-HLA binding affinity and by the difficulty to anticipate neoantigen immunogenicity *in silico*.

Bulk transcriptomics data can also be used to quantify different cell types of the TME through gene set enrichment analysis (GSEA) (19) or deconvolution. While GSEA can only asses the enrichment of cell types in a sample, deconvolution methods can quantitatively estimate relative cell fractions by considering the expression profile of bulk tumors as the “convolution” of cell-specific signatures (20). Recent tools like EPIC (21) and quanTIseq (22) are specifically developed for bulk RNA-seq data. The analysis of more than 8,000 tumor samples demonstrated that quanTIseq can be used to extract immunological scores with prognostic value and to monitor the pharmacological modulation of the immune contexture by anticancer drugs (22).

RNA-seq data can be used also to dissect the heterogeneity of T and B lymphocytes, which are equipped with an immensely diverse repertoire of receptors to be able to cope with a wealth of unpredictable antigens. When T cells encounter an antigen and get activated, they rapidly proliferate and differentiate, leading to a fast expansion of the T cell clone carrying the matching T-cell receptor (TCR). Computational methods like MiXCR (23) can analyze the mixed transcriptomes from sequenced cell populations to determine the diversity of the B- and T-cell receptors. Beside the prognostic value of lymphocyte-receptor diversity (23), immune repertoire profiling in tumors or blood can be used to predict or monitor anticancer immune responses triggered by ICBs (24–26), provided that enough lymphocytes are present in the samples.

Overall, RNA-seq produces very rich and unbiased datasets (i.e., not relying on sets of pre-selected markers) that enable different immuno-genomic analyses in parallel (13, 27). Its single-base resolution warrants analyses at the sequence level like the identification of fusion transcripts or the profiling of immune repertoires, which are not feasible from microarray data. Currently, most of computational approaches to dissect bulk measurements are dedicated to this data type due to the broad diffusion of sequencing technologies and by the establishment of large-scale coordinated sequencing efforts.

## The single-cell revolution in *Omics* technologies

**Figure 1:**
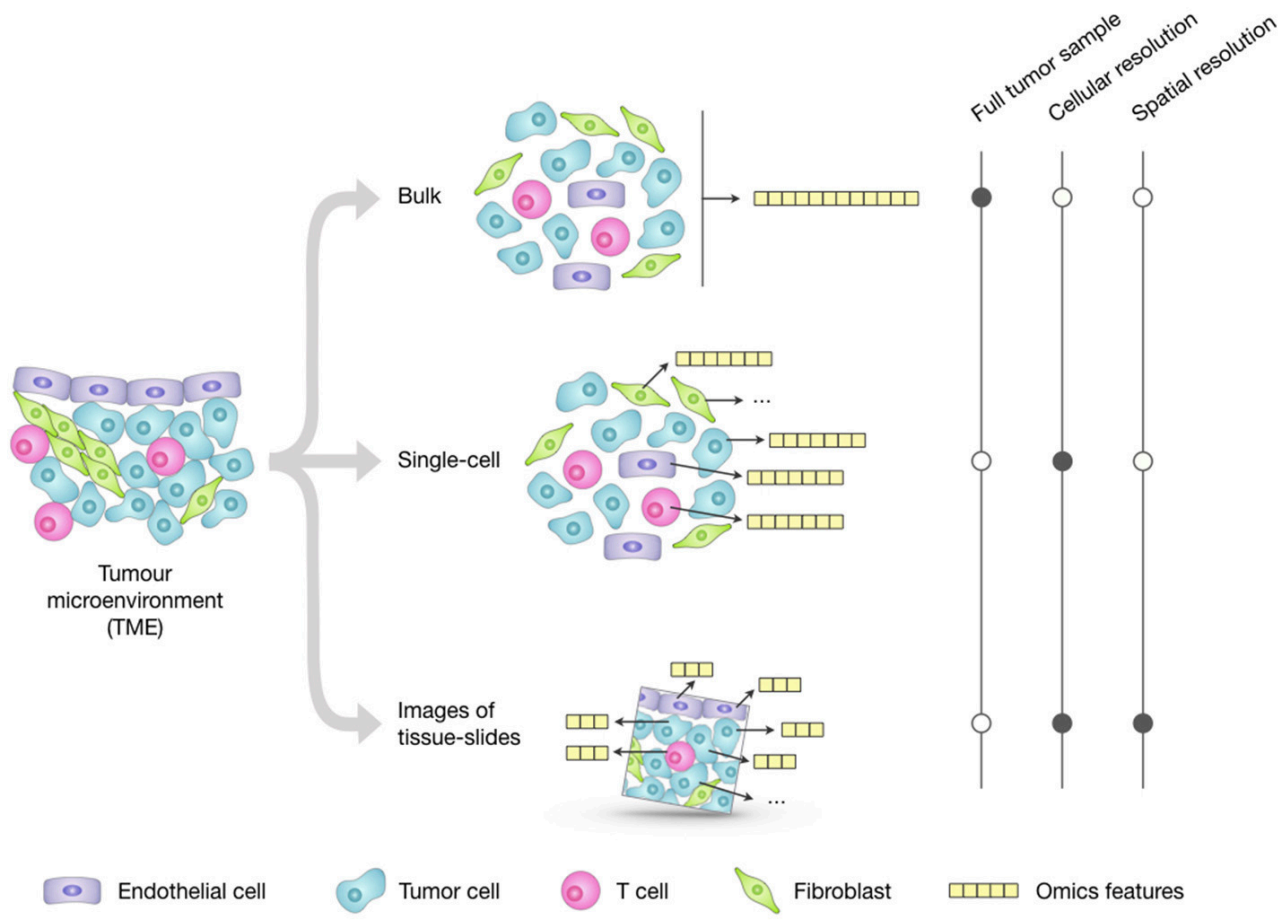
Overview of the main approaches for multi-omics profiling of the tumor microenvironment (TME). *Omics* datasets can be generated from bulk tumor samples; this approach is the most standardized and widely used and provides a high-throughput representation of the molecular features (e.g., genome, transcriptome, proteome) of the TME as a whole. Unlike the averaged representation provided by bulk approaches, single-cell technologies allow generating *omics* profiles of each individual cell; however, their costs and technical complexity currently limit the throughput in terms of number of features and total cells that can be assayed. Emerging imaging techniques can generate *omics* datasets from tumor-tissue slides that retain the cell spatial resolution; they have cellular or subcellular resolution but their throughput is significantly lower compared to the other two approaches and the resulting images only represent a restricted 2D snapshot of the tumor.

Profiling of bulk populations inevitably renders only a blended average portray that masks the peculiar contributions of individual cells. This limitation can be overcome thanks to new technologies that can generate different *omics* data at the single-cell level (Figure 1). The possibility to describe cell types and states at high resolution and granularity now provides the opportunity to catalog all human cells in health and disease (28).

Single-cell technologies can dissect intra- and inter-tumor heterogeneity and shed light on rare cells playing a role in cancer progression and invasion, like circulating tumor cells (CTC), cancer stem cells, and cells committed to epithelial-to-mesenchymal transition (EMT) (29). Single-cell DNA sequencing allows the investigation of cell-specific genetic variants and the reconstruction of tumor clonality and evolution *via* phylogenetic methods (29).

Single-cell RNA-seq (scRNA-seq) is leading the single-cell revolution in terms of both available technologies and pace of development, and currently allows the profiling of up to hundreds of thousands of cells in a single experiment and the interrogation of thousands genes (30–32). scRNA-seq is enabling the reconstruction of a high-resolution map of the TME in different cancer types (33–39) and, together with single-cell epigenomics, the characterization of the heterogeneity, plasticity, and functional diversity of the immune system (40, 41). Its unbiased nature is also opening up novel opportunities for the discovery of new immune cell subpopulations (42). scRNA-seq is currently not suited for the quantification of TME cell subtypes due to differences in single-cell dissociation efficiency that influence the representation of cell type proportions (39). However, the signatures reconstructed with fine granularity from scRNA-seq data can be used to inform deconvolution methods to make them able to quantify cell types with specific functional states (e.g., activated or dysfunctional CD8^+^ T cells) and to take into account the tissue and disease context.

Compelling advances have been also reported in the field of single-cell proteomics (43). Currently, most of these technologies, which can be broadly divided into cytometry- (44) and microfluidics-based (45) platforms, require the use of antibodies and allow assaying up to 50 proteins in hundreds of thousands of cells per sample. The number of measured molecules is likely to increase significantly in the future with further developments of DNA-labeled antibodies for higher multiplexing (46) and with the improvement of high-resolution mass-spectrometry (47).

Despite being constrained to the measurement of selected markers, proteomics holds the great advantage of directly measuring the functionality of the cells in the TME. Here, many functions are carried out by proteins, which mediate signal transduction, regulate transcription, are secreted as cytokines/chemokines, and are responsible to regulate cell migration and invasion. In particular, single-cell proteomics data from mass cytometry (CyTOF) have been used extensively to profile the tumor immune landscape (37, 48), to monitor how ICBs shape the population of immune cells and their function in responders and non-responders (49, 50), and to predict response to anti-PD-1 treatment from peripheral blood mononuclear cells (PBMCs) (49). Additionally, CyTOF holds great potential for the identification of neoantigens (51).

scRNA-seq and CyTOF technologies produce rich datasets that can be subjected to different computational analyses, including (but not limited to): unsupervised clustering of cell types, cell classification, pseudotemporal cell ordering, assignment, or correction of cell cycle stages, characterization of rare cells, identification of marker genes, and reconstruction of gene and signaling networks (43, 52–54).

Emerging technologies now also permit to simultaneously generate different *omics* data from the same cell (55) [e.g., proteomics and transcriptomics data (46, 56)], portraying the phenotypes of the different TME cells and revealing the molecular mechanisms that regulate their transcriptional programs. Additionally, integration of various *omics* data from different cells can provide further insights on the heterogeneity and organization of TME. For instance, scRNA-seq can be coupled with CyTOF data to confirm newly discovered cellular phenotypes.

Despite their great potential, a word of caution should be expressed regarding the experimental and analytical complexity of single-cell technologies. Computational analyses are challenged by high data dimensionality, high noise and absence of biological replicates *per se*, and low coverage that can result in extreme data sparsity (57, 58). These issues have already sparked a fast-paced development of new technologies and computational tools, but further efforts are required to reach full maturity and standardization.

## Toward multi-cellular modeling of the tumor microenvironment

The organization of cells in the TME plays an important role as individual cells are strongly affected by their surroundings. Unlike bulk and single-cell technologies, imaging techniques of tumor-tissue slides allow the spatial localization of tumor, immune, and stromal cells, blood vessels, and ECM, and can assay expressed genes and proteins at subcellular resolution (Figure 1).

The best-established imaging techniques for protein phenotyping of tumor-tissue slides are immunohistochemistry (IHC) and immunofluorescence (IF). Multiplexed IHC by iterative staining of single slides or multispectral imaging (59–61), when combined with software for cell segmentation and marker-based classification, portrays the cellular architecture of the TME, but can consider only a limited number of markers. Emerging antibody-based imaging techniques [reviewed in (62, 63)], like imaging mass cytometry (IMC) (64) and multiplexed ion beam imaging (MIBI) (65), can produce omics-like data through the quantification of up to 40 markers. However, they require a longer measurement time, limiting the size of the slide that can be imaged.

Moving toward multi-omics technologies, Schulz and colleagues recently adapted IMC to measure transcriptomics and proteomics markers on the same cell using metal-labeled oligonucleotides and antibodies, respectively (66). Interestingly, they found that HER2 mRNA and protein expression correlate well at the population level, but not at the single-cell level. Moreover, they showed that cells expressing CXCL10, a chemokine favoring the recruitment of T cells in the TME, organize in clusters. These so-called *tissue motifs* have been identified also by studying the spatial organization of stem cells niches and tumor-infiltrating immune cells (62, 67) and highlight the importance of considering spatial patterns for a better understanding of tissue biology. Another approach to look at cell-cell interactions is the investigation of intercellular signaling. A recent effort in this direction has been the reconstruction of an intercellular communication map of ovarian adenocarcinoma using bulk proteomics and transcriptomics data from tumor cells, tumor-associated T cells, and macrophages (68). Transcriptomics data have been also used to study intracellular regulation, for example to understand the importance of CD4^+^ T cells in supporting the effector function of cytotoxic T cells (69).

Systems biology approaches can be used to describe the different cell types of the TME, together with their intra- and inter- cellular regulatory mechanisms, leveraging on mathematical models to provide a holistic view of all these components. Mathematical models can unravel mechanisms of tumorigenesis and make predictions, which can be experimentally validated (70, 71). Dynamic models have been used to describe intracellular signaling to understand resistance and suggest personalized therapies (72–74), and they can be extended to study also extra-cellular signaling (75). Cell-cell interactions can be modeled considering individual cells as agents, thus providing a spatial description of the system (76), or as black-boxes with a certain input-output behavior (77). Mathematical models have been used to understand cellular interactions, to test *in silico* the effect of different therapeutic interventions, and to investigate tumor initiation and progression (70).

Given the progress of technologies for profiling spatially-resolved RNA and protein expression data, new computational methods are strongly required to study cellular networks from subcellular markers taking into account spatial information (78, 79). Current approaches are mainly data-driven, but they will extend to mechanistic models incorporating prior knowledge on cell-type-specific intra-cellular pathways and ligand-receptor interactions (80).

## Implications for cancer therapy and immunotherapy

All the new insights brought about by the immuno-oncology revolution and by the fast development of *omics* technologies highlight the need to revise the concept of intra-tumor heterogeneity from a broader perspective and to consider its implications for cancer therapy and immunotherapy.

The first layer of heterogeneity of the TME regards cancer cells. Genetic features alone neither fully recapitulate tumor-cell diversity and dynamicity, nor predict individual drug response (81, 82). The integration of other *omics* data can shed light on the evolution of tumor-cell phenotypes during tumor progression and therapy and to overcome the current limits of precision medicine (83, 84). Moreover, the characterization of tumor neoantigens can open new avenues for personalized vaccination (85) but, to see their clinical translation, these approaches need to be optimized in terms of precision and time efficiency. Non-malignant cells of the TME, like immune cells and CAFs, introduce a second layer of cellular heterogeneity, as their action can sustain or counteract tumor progression. Anticancer immune responses can be boosted employing agents that deplete immunosuppressive cells, increase the infiltration of CD8^+^ T cells or restore their effector function. A third layer of heterogeneity is given by the interaction between cells, which is influenced by their expressed/secreted molecules, carried antigens, or receptor diversity. Regarding the interplay between tumor and immune cells, accumulating evidence suggests that most of conventional and targeted therapies owe their efficacy to the induced reactivation of the immune system (86), and that oncogenic pathways are responsible for resistance (2) and modulation of the immune system in the TME (87). By disentangling the different layers of heterogeneity of the TME, we can gain mechanistic rationale to unlock the synergistic potential of ICBs and targeted agents (88).

A pressing need in cancer immunotherapy is the identification of predictive biomarkers because only a limited fraction of patients responds to ICBs. Tumor mutational load, microsatellite instability, and PDL1 expression have been associated with response to blockade of the PD1/PDL1 axis, but no marker provides a clear-cut separation of responders and non-responders (89). Recently, there have been several attempts to develop predictors of the response to ICBs (90–92), but their optimization and validation are currently curtailed by the limited public data available. The identification of biomarkers becomes even more critical for combinatorial therapy. The number of clinical trials for combination therapies based on anti-PD-1/PD-L1 was estimated to be 1,100 worldwide in 2017, but their validity has been questioned due to the lack of rationale for patient stratification (93).

One approach for the identification of more informative biomarkers could be the generation of multi-omics data from longitudinal samples. In this direction, Riaz et al. (25) used RNA-seq, whole-exome, and TCR sequencing to investigate the evolution of the TME in melanoma patients treated with anti-PD1. However, a more holistic view of the TME as a multicellular system is essential to rationally design clinical trials and inform on predictive, dynamic biomarkers. Given the dynamic nature of the immune system, systems biology approaches can improve personalized immunotherapy (94) calling for collaboration between experimental and computational scientists (95, 96). Computational system models can incorporate single-cell and bulk multi-omics data and will help to understand how the TME components operate and to predict the effect of therapies on the system as a whole. Moreover, mathematical models can be used for *in silico* screening of immunotherapies and combination treatments, providing personalized recommendations (97).

Since tumors are complex systems, prediction of response to therapy is far from trivial despite the availability of rich multi-omics datasets. A complementary approach to profiling the TME for precision medicine is the use of functional screening to directly assay the response of the tumor to drug perturbations (98). In this respect, mouse models and tumor organoids are powerful approaches to test a large number of perturbations on *ex vivo* tumor cultures. Advances in the co-culturing of organoids and immune cells might enable to functionally screen ICBs and to select tumor-reactive T cells to be used for immunotherapy (99, 100). Alternatively, miniaturized volumes with droplet-based microfluidics technologies can be used to screen drugs directly on tumor biopsies (101, 102) and can be further adapted to identify synergistic partners for ICBs.

## Conclusions and future perspectives

Thanks to the scientific insights derived from the recent advancements in the immuno-oncology field, fostered also by the pressing development of *omics* technologies, the investigation of the TME is gaining momentum. It is now clear that tumors are not uniform masses of malignant cells, but multifaceted ecosystems characterized by: (1) profound cellular heterogeneity, (2) dynamicity and plasticity, and (3) complex cell-cell interactions.

**Figure 2:**
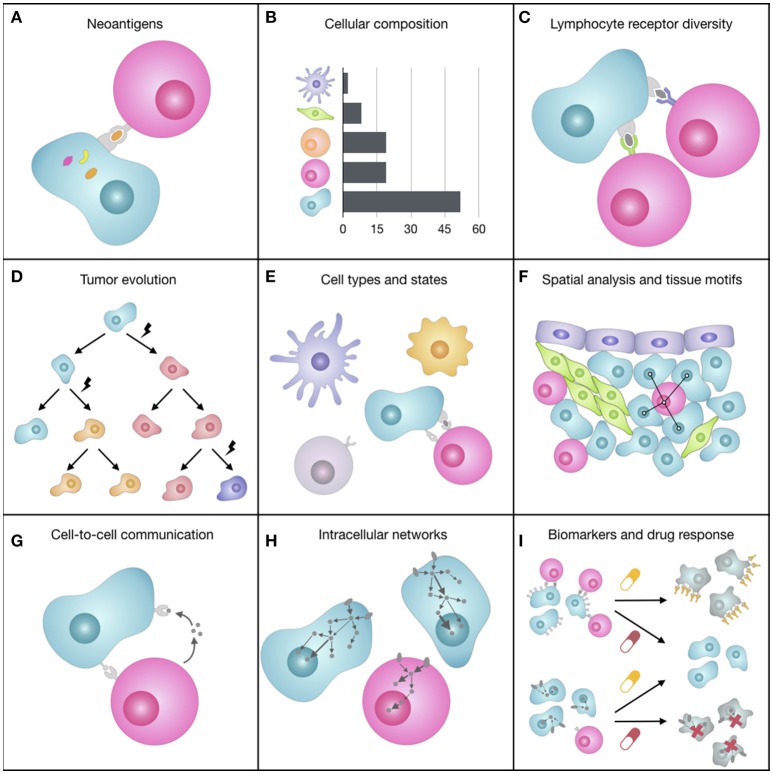
Representation of some of the facets of the tumor microenvironment (TME). **(A)** The expression of mutated genes can generate tumor-specific neoantigens, i.e., peptides bound to the tumor cell HLAs that can be recognized by T cells and elicit an immune response. **(B)** The quantification of the different cell types of the TME, which can have pro- or anti-tumorigenic roles, can provide prognostic, and predictive markers for immunotherapy. **(C)** The immense diversity of lymphocyte receptors, which can be different from lymphocyte to lymphocyte, allows the immune system coping with a wealth of unpredictable antigens and varies greatly depending on spontaneous or therapy-induced anticancer immune responses. **(D)** During tumor progression, cancer cells accumulate somatic mutations that increase intra-tumoral heterogeneity and can change cell fitness and response to drugs. **(E)** The TME is composed of various cell subtypes (e.g., CD4+ and CD8+ T cell), which are in turn characterized by different functional orientations (e.g., naïve, effector, memory CD8+ T cell) and states (e.g., activated, anergic, exhausted CD8+ T cell). **(F)** The spatial organization of cells, such as cell neighbors (e.g., proximity of tumor and immune cells) or cellular patterns (i.e., tissue motifs, such as stem-cell niches), reflects biological processes at the tissue level. **(G)** Cells constantly exchange signals with surrounding cells by secreting molecules (e.g., cytokines, chemokines, growth factors) or by direct ligand-receptor binding on the cell surfaces (e.g., immune checkpoints). **(H)** When ligands bind to cell receptors, the cell responds by processing this signal through a complex signal transduction network that transmits information to the nucleus, where transcription factors regulate the transcriptional response of the cell. **(I)** Most of cancer therapies, such as immunotherapy with immune checkpoint blockers or targeted therapy, act on molecules responsible for inter- and intra-cellular communication that are deregulated in cancer, trying to restore the normal behavior of the cells.

This broader vision of the TME that is emerging with the support of bioinformatics (Figure 2) offers the opportunity to redefine the “precision oncology” paradigm and to eventually augment its curative potential, still hampered by a too narrow, genomic-focused implementation (83, 84). We envision that computational methods for the systematic integration of multi-omics data [reviewed in (103, 104)] will be tailored to reveal features of the TME underlying differential patient responses. Moreover, further therapeutics advancements are expected from the rational combination of ICBs with targeted agents, which are likely to act synergistically and extend the long-term clinical benefits of immunotherapy to a larger fraction of patients (88).

We are now at a crossroad to unlock the potential of precision immuno-oncology, which can lead to the discovery of predictive biomarkers for immunotherapy, understanding of mechanisms of resistance, and design of combination therapies with higher clinical success. To this end, we need to embrace a holistic approach in the investigation of the different facets of the TME in order to master its modulation and finally achieve an effective control of tumor growth. We envision that this new paradigm in precision medicine will lay its foundations on the integration of different *omics* data, and that bioinformatics and system biology will play a central role for the extraction of novel mechanistic insights on the TME that can guide the immuno-oncology field.

## Author contributions

All authors listed have made a substantial, direct and intellectual contribution to the work, and approved it for publication.

### Conflict of interest statement

The authors declare that the research was conducted in the absence of any commercial or financial relationships that could be construed as a potential conflict of interest. The handling editor declared a shared affiliation, though no other collaboration, with one of the authors FF.

## Abbreviations

CAF: cancer associated fibroblast
CTC: circulating tumor cells
CTLA-4: cytotoxic T-lymphocyte-associated protein 4
CyTOF: cytometry by time of flight
ECM: extracellular matrix
EMT: epithelial-to-mesenchymal transition
GSEA: gene set enrichment analysis
HLA: human leukocyte antigen
ICB: immune checkpoint blocker
IF: immunofluorescence
IHC: immunohistochemistry
IMC: imaging mass cytometry
MDSC: myeloid-derived suppressor cell
MIBI: multiplexed ion beam imaging
PBMC: peripheral blood mononuclear cell
PD-1: programmed cell death protein 1
PD-L1: programmed cell death-ligand 1
RNA-seq: RNA sequencing
scRNA-seq: single-cell RNA sequencing
TCR: T-cell receptor
TME: tumor microenvironment
T_reg_ cell: regulatory T cell.
